# A systematic review of the prevalence of parental concerns measured by the *Parents’ Evaluation of Developmental Status (PEDS)* indicating developmental risk

**DOI:** 10.1186/1471-2431-14-231

**Published:** 2014-09-13

**Authors:** Susan Woolfenden, Valsamma Eapen, Katrina Williams, Andrew Hayen, Nicholas Spencer, Lynn Kemp

**Affiliations:** Department of Community Child Health, Sydney Children’s Hospital Network, High St Randwick NSW 2031, Sydney, Australia; School of Psychiatry, University of New South Wales, Sydney, Australia; Royal Children’s Hospital and Murdoch Children’s Research Institute, University of Melbourne, Melbourne, Australia; School of Public Health and Community Medicine, University of New South Wales, Sydney, Australia; Warwick Medical School, University of Warwick, Coventry, UK

**Keywords:** Prevalence, Parental concerns, Parents Evaluation of Developmental Status (PEDS), Risk factors, Developmental risk, Child health

## Abstract

**Background:**

Parental concerns about their children’s development can be used as an indicator of developmental risk. We undertook a systematic review of the prevalence of parents’ concerns as an indicator of developmental risk, measured by the *Parents’ Evaluation of Developmental Status (PEDS)* and associated risk factors.

**Methods:**

Electronic databases, bibliographies and websites were searched and experts contacted. Studies were screened for eligibility and study characteristics were extracted independently by two authors. A summary estimate for prevalence was derived. Meta-regression examined the impact of study characteristics and quality. Meta-analysis was used to derive pooled estimates of the impact of biological and psychosocial risk factors on the odds of parental concerns indicating high developmental risk.

**Results:**

Thirty seven studies were identified with a total of 210,242 subjects. Overall 13.8% (95% CI 10.9 -16.8%) of parents had concerns indicating their child was at high developmental risk and 19.8% (95% CI 16.7-22.9%) had concerns indicating their child was at moderate developmental risk. Male gender, low birth weight, poor/fair child health rating, poor maternal mental health, lower socioeconomic status (SES), minority ethnicity, not being read to, a lack of access to health care and not having health insurance were significantly associated with parental concerns indicating a high developmental risk.

**Conclusions:**

The prevalence of parental concerns measured with the *PEDS* indicating developmental risk is substantial. There is increased prevalence associated with biological and psychosocial adversity.

**Trial registration:**

PROSPERO Registration: CRD42012003215.

**Electronic supplementary material:**

The online version of this article (doi:10.1186/1471-2431-14-231) contains supplementary material, which is available to authorized users.

## Background

Children at developmental risk, are those who have significant problems in at least one area of their development (e.g., motor, language, self-help, social, cognitive, behavioural) [1]. They include children who may be at risk of having a developmental disorder, or children who are functioning on the lower end of normal who may go on to struggle with the literacy, numeracy and socio-emotional demands of school and life [1]. Adverse childhood experiences including socioeconomic disadvantage, poor parental mental health, lack of stimulating early childhood experiences, and lack of access to services can contribute to developmental risk [2–6].

In order to develop a comprehensive public health response to optimise early childhood development, it is helpful if we are able to quantify the state of child development from a population perspective. Although not a comprehensive developmental assessment, measuring parental concerns about their children’s development can be done in a quick, standardised, systematic manner and has been used to estimate level of developmental risk in the general population and to identify high risk subpopulations [7, 8]. In addition, eliciting and addressing parental concern is a key component in the family centred practice of detecting individual children at developmental risk in well child health care so that they may have timely referral on for assessment and early intervention prior to starting school [9–12]. The *Parents’ Evaluation of Developmental Status* (*PEDS*) is a 10 − item parent completed standardised questionnaire, which has been used to elicit parental concerns around child development for children aged less than 8 years in populations, communities and clinical samples. The *PEDS* open ended questions cover expressive and receptive language, fine motor, gross motor, behaviour, socialisation, self care, and learning [13]. An estimate of developmental risk as high, moderate, low or no risk is derived from the parental concerns recorded and a clinical pathway is recommended. The *PEDS* has a sensitivity of 91-97% and specificity of 73-86% in recent validation studies from the USA for the accuracy of parental concerns in detecting children at high and/or moderate developmental risk [14]. The *PEDS* has been found to be useful in vulnerable disadvantaged populations, high, middle and low income countries, and has been translated in multiple languages [14, 15]. There is also a modified version of the *PEDS*, the *Survey PEDS* which has 12 close-ended questions that does not allow for further discussion of parental concerns and clinical decision making around these. It is less well validated than the clinical form of the PEDS but is used in telephone population surveys [7, 14, 16–18].

In order to better understand the current worldwide prevalence of parental concern measured by the *PEDS* that indicate developmental risk and associated risk factors, we undertook a systematic review to synthesize the available international evidence.

## Methods

### Search strategy

A protocol was developed and registered with the University of York Centre for Reviews and Dissemination (PROSPERO) on 6/11/2012 and updated on the 13/02/2014, registration number CRD42012003215(http://www.crd.york.ac.uk/PROSPERO/index.asp).

A systematic search of the literature was undertaken using the following inclusion criteria: primary observational studies (cohort study, cross-sectional studies) in geographically defined population or a community sample (including samples from primary health care services) of children aged under 8 years using the *PEDS* [15] with available prevalence data (Additional file 1). Studies using the modified “*Survey PEDS*” were also included in this review [14]. Electronic databases searched were Web of Science and Google Scholar, PubMed (Nov 2012), EMBASE (Nov 2012), Medline (Nov 2012), Psychinfo (Nov 2012), Global Health (Nov2012) CINAHL (Nov 2012), the Cochrane Library (Nov 2012), LILACS (Nov 2012), ERIC (Nov 2012), and Proquest (Nov 2012). Secondary searches of citations in review articles, requests to experts in the field and additional searches of the USA based *PEDS*test and RCH *PEDS* website for key studies were undertaken. Advice from the Cochrane Child Development, Psychosocial and Learning Groups was sought regarding search terms which were specific for early child development, developmental risk and the *PEDS*. There were no language limitations. Studies using specific clinical samples, for example, neonatal intensive care graduates or with participants who had a known developmental disorder were excluded.

The study titles, abstracts and full papers of “potentially relevant articles” were reviewed independently by two authors (SW&VE). Disagreements about inclusion were resolved through consensus and discussion with a third author (KW). Study characteristics, prevalence, and risk factors, were extracted independently by SW and VE on a data extraction form that was piloted and modified prior to use. Where insufficient data were reported, study authors were contacted. If no reply was forthcoming or full data not made available, data were included in analysis where possible. Methodological quality was assessed independently by SW and VE based on a validity of the study methods (design, sampling frame, sample size, outcome measures, measurement and response rate), interpretation of the results and applicability of the findings [19], a score of 6 or greater was rated by the reviewers as high quality.

### Statistical analysis

#### Prevalence

Estimates of the prevalence of parental concerns on the *PEDS* indicating moderate or high risk with corresponding 95% confidence intervals were extracted from each study. If the confidence intervals were not provided, these were calculated using the Agresti and Coull method [20]. For longitudinal studies, cross-sectional estimates of prevalence were used to extract prevalence data at the first time point. We used an exact likelihood approach to obtain pooled estimates of prevalence. We used metaregression, a regression method that allows the examination of study-level factors on prevalence with the following pre-specified variables on prevalence: sample type; type of *PEDS*; study purpose; study quality; study age group, publication type and country income [21].

### Risk factor analysis

We conducted a meta-analysis for risk factors for having parental concerns on the *PEDS* indicating high versus low/no developmental risk. We extracted odds ratios and 95% confidence intervals from each study. If odds ratio (OR) with a 95% confidence interval was not provided, we calculated the odds ratio and 95% confidence interval. We extracted adjusted odds ratios when possible, but we were unable to calculate these for studies in which they were not provided. We obtained pooled estimates of unadjusted odds ratios (uOR) using meta-analysis with random effects. Where studies presented adjusted odds ratios (aOR) for similar child and family variables these were combined in a separate meta-analysis.

### Investigation of heterogeneity

For all meta‒analyses and meta‒regressions of prevalence, we modelled within-study variability using the binomial distribution [21]. We then examined heterogeneity through meta-regression models, as described in previous systematic reviews of prevalence [22]. We quantified the reduction in the between study variance from the inclusion of the study characteristics compared to the ‘base’ model (i.e., the model of prevalence without any covariates). This provides an estimate of the proportion of heterogeneity that is explained by that characteristic. For our meta-analysis of risk factors, the degree of heterogeneity was investigated by estimating the I^2^ statistic (which describes variation in the summary effect due to genuine variation rather than a sampling error as a percentage, a low I^2^ indicates low heterogeneity and high I^2^ indicates significant between study variability) and visual inspection of forest plots [22].

## Results

### Studies identified

**Figure 1:**
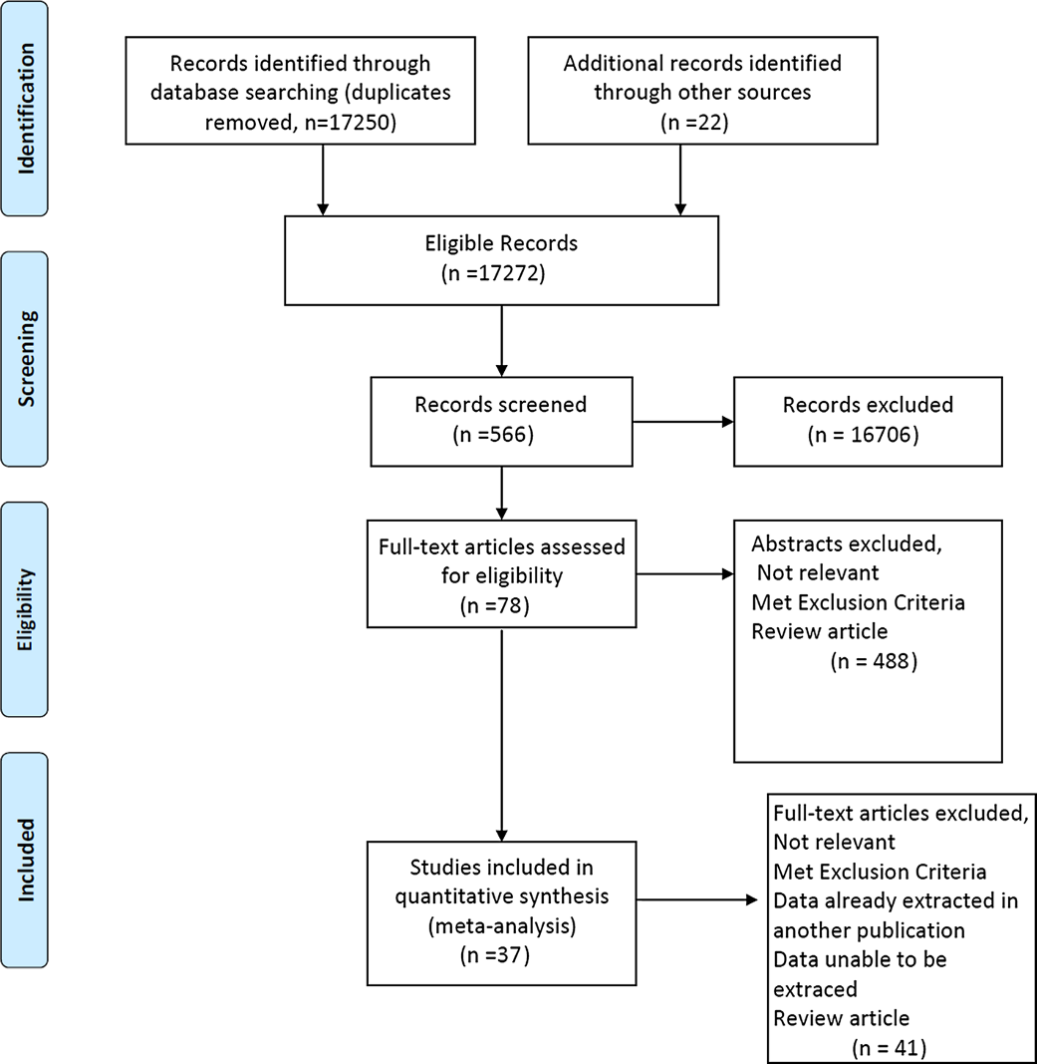
**Search flow chart.**

The search strategy identified 17,272 titles (excluding duplicates). Seventy-eight articles underwent a text screen and 41 of these were excluded (Figure 1) [23].

### Included studies

The prevalence estimates of the 37 included studies are listed in Table 1 [7, 13–18, 24–56]. Twenty three studies were published in peer review journals, and the remainder were government/university reports, unpublished abstracts available on the *PEDS*test website, online population survey data and data from the *PEDS* Standardisation Manual (2013). There was one longitudinal cohort with data available on samples at two time points three years apart [39, 40, 57]. All other studies were cross sectional.

**Table 1:** Included studies characteristics and prevalence rates*

First author	Country	Age (months)	Sample size	Quality score/8	High risk% (95% CI)	Moderate risk% (95% CI)	High and moderate risk% (95% CI)	Low/no risk% (95% CI)
Armstrong [15]	Australia	0-95	246	3	11.4 (8.0-16.0)	21.9 (17.2-27.6)	33.3 (27.7-39.5)	66.7 (60.5-72.3)
Bethell [29]	USA	10-71	22883	8	9.6(9.2-10.0)	15.9 (15.5-16.4)	25.5 (25.0-26.1)	74.5 (73.9-75.0)
CHIS [50]	USA	4-60	2884	7	25.6 (23.6-27.5)	17.4 (15.6-19.2	43.0 (41.2-44.8)	57.0 (55.2-58.8)
CHIS [49]	USA	4-60	3029	7	19.9 (18.3-21.5)	18.0 (16.4-19.6)	37.9 (36.2-39.2)	62.2 (60.4-63.9)
CHIS [48]	USA	4-60	3058	7	26.3 (24.5-28.2)	18.3(16.7-19.9)	44.7 (72.9-46.4)	55.3 (53.6-57.1)
CHIS [47]	USA	4 to 60	3096	7	20.1 (17.6-22.5)	19.7 (17.4-22.0)	39.8 (38.1-41.5)	60.2 (58.5-61.9)
Chuan [24]	Malaysia	12-72	86	2	26 (17.5-35.8)	NA	NA	17.0 (10.8-27.0)
Coghlan [28]	Australia	18-69	262	3	9.2 (6.2-13.3)	18.7 (14.4-23.9)	27.9 (22.8-33.6)	72.1 (66.4-77.2)
Davies [25]	UK	0-24	76	5	2.6 (0.2-9.8)	13.2 (7.2-22.8)	15.8 (9.2-25.8)	84.2 (74.2-90.8)
Glascoe [32]	USA	24-84	408	5	NA	NA	34.6 (30.1-39.3)	65.4 (60.7-69.9)
Glascoe [58]	USA	3-93 (mean 46.5 SD 21.8)	771	5	11.0 (9–13.4)	26.0 (23.0-29.2)	37.0 (33.6-40.4)	63.0 (59.6-66.4)
Glascoe [33]	USA	mean 36	257	4	41.0 (35.0-47.0)	40.0 (34.3-46.2)	81.0 (75.6-85.3)	19.0 (14.7-24.4)
Glascoe [33]	USA	mean 36	744	4	23.0 (20.1-26.2)	26.0 (22.9-29.2)	49.0 (45.4-52.5)	51.0 (47.5-54.7)
Glascoe [14]	USA	0.3-95 (mean 26 SD 20.6)	47531	6	4.5 (4.3-4.7)	13.7 (13.4-14.0)	18.2 (17.9-18.6)	81.8 (81.5-82.1)
Gustawan [59]	Indonesia	3-12	170	3	NA	NA	31.0 (24.2-37.9)	69.0 (62.1-75.8)
Ibironke [56]	USA	6-71 (mean 38.5 SD 18.4)	2381	7	NA	NA	21.4 (19.8-23.1)	78.6 (76.9-80.2)
Kiing [41]	Singapore	1-83	1806	3	7.5 (6.4-8.8)	26.0 (24.1-28.1)	33.5 (31.4-35.7)	66.0 (64.3-68.6)
Kosht-Fedyshin [42]	Tanzania	0-60	20	4	35.0 (18.1-56.9)	0.0	35.0 (18.1-56.9)	65.0 (43.1-81.9)
Limbos [43]	Canada	12-60	331	5	13.9 (10.6-18.1)	39.6 (34.5-45.0)	53.5 (48.1-58.8)	46.5 (41.2-51.9)
Malhi [44]	India	24-60	79	2	NA	NA	39.2 (29.2-50.3)	60.8 (49.7-70.8)
Matibag [45]	Philippines	24-60 (mean 53.6)	283	2	15.0 (11.2-19.5)	NA	NA	NA
McGookin [35]	USA	9-24	385	3	5.2(3.4-8.0)	17.4 (13.9-21.5)	22.6 (18.7-27.1)	77.4 (73.0-81.3)
Ng [18]	Canada	0-83 (mean 46.1)	419	6	9.3 (6.9-12.5)	18.9 (15.4-22.9)	28.2 (24.1-32.7)	72.0 (67.3-75.9)
NSCH (2011/2012) [16]	USA	4-60	28540	8	77.0 (10.1-11.9)	15.2 (14.3-16.1)	26.2(25.7-26.7)	73.8 (72.7-75.0)
Oreto [46]	Philippines	0-84 (means 53)	318	4	15.1 (11.6-19.5)	17.0 (13.3-21.5)	32.1 (27.2-37.4)	67.9 (62.6-72.8)
Palarca [51]	Philippines	0.5-96 (means 52.6)	421	3	9.0 (6.6-12.2)	5.0 (3.3-7.6)	14.0 (11.0-17.7)	86.0 (82.3-89.0)
Restall (2009) [52]	Canada	60	290	6	13.1 (9.7-17.5)	32.4 (27.3-38.0)	45.5 (39.9-51.3)	54.5 (48.7-60.1)
Rose-Jacobs [37]	USA	4-36	2010	5	13.8 (12.4-15.4)	NA	NA	NA
Roux [26]	USA	<60	2845	3	28.2 (26.6-29.9)	27.5 (25.9-29.2)	55.7 (53.9-57.5)	44.3 (42.5-46.1)
Sarmiento Campos [31]	Spain	6-42	1089	3	8.5 (7.0-10.4)	23.0 (20.7-25.7)	31.6 (28.9-34.4)	68.4 (65.6-71.1)
Sices [38]	USA	9-31 (means 17.6 SD 6.1)	60	2	26.7 (17.1-39.1)	10.0 (4.4-20.6)	36.7 (25.6-49.4)	63.3(50.6-74.4)
Stevens [7]	USA	4.35	2068	6	23.4 (21.6-25.3)	24.9 (23.1-26.8)	48.3 (46.2-50.5)	51.7 (49.5-53.9)
Theeranate [53]	Thailand	0-72	216	3	NA	NA	4.2 (2.1-7.9)	95.8 (92.1-97.9)
Tough [40]	Canada	Mean 38 (SD 8)	792	4	10.8 (8.9-13.2)	30.2 (27.1-33.5)	41.0 (37.7-44.5)	59.0 (55.5-62.3)
VSEHQ (2008) [54]	Australia	60-83	54602	6	7.2 (7.0-7.4)	16.5 (16.2-16.8)	23.7 (23.3-24.0)	76.3 (76.0-76.7)
Wake [55]	Australia	63.4-90	853	3	NA	NA	35.0 (31.9-38.3)	65.0 (61.7-68.1)
Zuckerman [34]	USA	<72	24933	7	NA	NA	22.4 (21.9-23.0)	77.6 (77.05-78.1)

*quality rating system as per quality rating tool developed by Public Health Agency in Canada [19].

Fifteen studies used the *PEDS* as a research tool to measure prevalence of developmental risk of which 12 were population surveys in high income countries and three were community samples. The remaining studies used the *PEDS* as a developmental surveillance tool in primary health care and early childhood education/early primary school settings [14, 24–28, 31–33, 35, 38, 41–46, 51, 53, 58, 59]. Eight of the studies were conducted in low and middle income countries [24, 42, 44–46, 51, 53, 59] and two studies were in socioeconomically disadvantaged communities in the USA [33].

Study sample sizes ranged from 20 to 54602 (median = 467). There were 210,242 subjects in total. Ages ranged from less than 1 month to 7 years and 11 months consistent with the age range for administration of the *PEDS*. Twenty seven of the studies used translated versions of the *PEDS* for at least part of their sample.

### Study quality

Quality scores varied between studies (Table 2). Only 13 studies met 6 or more criteria and thus were deemed of high quality [7, 14, 16, 18, 29, 34, 47–50, 52, 54, 56]. Key areas of potential bias were lack of random selection of the sample (22/37), a biased sampling frame (20/37), less than 300 participants (11/37), less than 70% response rate and refusers not described (11/37); confidence intervals not given for prevalence results and lack of subgroup analysis (31/37).

**Table 2:** Quality assessment of included studies*

First author	Year	Random sample or whole population	Unbiased sampling frame (i.e. census data)	Adequate sample size (>300 subjects)	Measures were the standard	Outcomes measured by unbiased assessors	Adequate response rate (70%) and refusers described	Confidence intervals and subgroup analysis	Study subjects descirbed	Quality risk rating/8
Bethell	2011	1	1	1	1	1	1	1	1	8
NSCH	2011/2012	1	1	1	1	1	1	1	1	8
CHIS	2003	1	1	1	1	1	1	0	1	7
CHIS	2005	1	1	1	1	1	1	0	1	7
CHIS	2009	1	1	1	1	1	1	0	1	7
CHIS	2007	1	1	1	1	1	1	0	1	7
Ibironke	2011	1	1	1	1	1	0	1	1	7
Zuckerman	2009	1	1	1	1	1	0	1	1	7
Glascoe	2013	1	1	1	1	1	0	0	1	6
Stevens	2006	1	1	1	0	1	0	1	1	6
Ng	2010	1	1	1	1	1	0	0	1	6
Restall	2009	1	1	0	1	1	0	1	1	6
VSEHQ	2008	1	1	1	1	1	0	0	1	6
Davies	2009	1	1	0	1	1	1	0	0	5
Glascoe	1999	0	1	1	1	1	0	0	1	5
Glascoe	1997	0	1	1	1	1	0	0	1	5
Rose Jacobs	2008	0	0	1	1	1	1	0	1	5
Glascoe	2010	0	0	1	1	1	0	0	1	4
Glascoe	2010	0	0	1	1	1	0	0	1	4
Kosht-Fedyshin	2006	1	0	0	1	1	0	0	1	4
Limbos	2011	0	0	1	1	1	1	0	1	5
Oreto	2010	0	0	1	1	1	0	0	1	4
Tough	2008	0	0	1	1	1	0	0	1	4
Armstrong	2008	0	1	0	1		0	0	1	3
Campos	2010	0	0	1	1	1	0	0	0	3
Coghlan	2003	0	0	0	1	1	0	0	1	3
Kiing	2012	0	0	1	1	1	0	0	0	3
McGookin	2011	0	0	1	1	1	0	0	0	3
Palarca	2008	0	0	1	1	1	0	0	1	3
Roux	2011	0	0	1	1	1	0	0	0	3
Theeranate	2005	0	0	0	1	1	0	0	1	3
Wake	2005	0	0	1	1	1	0	0	0	3
Gustawan	2010	0	0	0	1	1	0	0	1	3
Chuan	2012	0	0	0	1	1	0	0	0	2
Mahli	2002	0	0	0	1	1	0	0	0	2
Matibag	2008	0	0	0	1	1	0	0	0	2
Sices	2009	0	0	0	1	1	0	0	0	2

*Quality rating system as per quality rating tool developed by Public Health Agency in Canada [19].

### Prevalence of developmental risk

**Figure 2:**
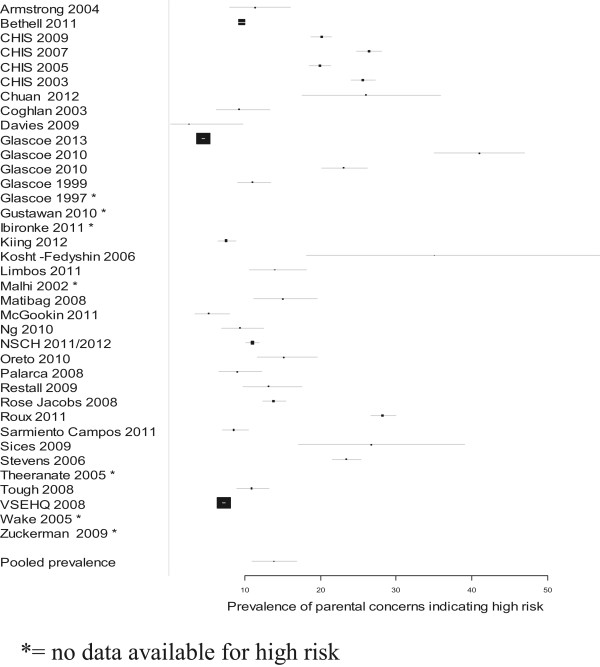
**Prevalence of parental concerns indicating high developmental risk.**

The pooled estimate of the prevalence of parental concern on the *PEDS* indicating high developmental risk was 13.8% (95% CI 10.9-16.8%), meaning that almost 14% of parents raised concerns associated with a high risk for developmental problems (Figure 2). The pooled estimate of for moderate developmental risk was 19.8% (95% CI 16.7-22.9%). The pooled estimate for high or moderate developmental risk was 31.5%(95% CI 27.0-36.0%), meaning that more than 31% raised concerns associated with either high or moderate risk of developmental problems.

Meta-regression was conducted for study characteristics (Table 3). Peer reviewed publications had a significantly lower level of parental concerns indicating high developmental risk on the *PEDS* than unpublished sources (abstracts, reports and population survey data available on the internet). This variable contributed to 19% of the overall variance between studies. For the report of parental concerns on the *PEDS* indicating moderate developmental risk, studies done in high income countries reported a significantly higher rate than those done in low and middle income countries. This variable contributed to 29% of the overall variance between studies. All other variability in study characteristics did not have an impact.

**Table 3:** **Metaregression of included studies**

Study characteristics	Prevalence of high risk (%, 95% CI)	P value	Prevalence of moderate risk (%, 95% CI)	P value
**All studies**	13.8 (10.9,16.8)		19.9 (16.8,23.1)	
**Sample type**				
Population survey	13.5 (8.8,18.1)	0.86	18.7 (14.3,23.1)	0.47
Community sample	14.0 (10.2,17.8)		21.0 (16.7,25.2)	
**Type of PEDS**				
Survey PEDS	17.9 (10.9,24.9)	0.15	20.6 (16.9,24.3)	0.50
Clinical PEDS	12.6(9.6,15.7)		18.3 (12.9,23.7)	
**Study purpose**				
Population risk measure	14.5 (9.7,19.2)	0.71	20.1 (15.4,24.9)	0.92
Developmental surveillance tool	13.3 (9.6,17.1)		19.8 (15.6,24.0)	
**Study quality**				
High quality	13.5 (8.8,18.1)	0.86	18.7 (14.3,23.1)	0.47
Medium/low quality	14.0 (10.2,17.8)		21.0 (16.7,25.2)	
**Study age group**				
3 years and under	14.5(8.9,20.2)	0.76	22.9(16.7,29.1)	0.25
Includes over 3 years	13.5(10.1,16.9)		18.9 (15.4,22.3)	
**Publication type**				
Peer review/Government report	11(8.1,14.0)	0.013	21.1(16.9,24.4)	0.39
Abstracts/website/manuals	18.0(13.0,22.9)		18.4(14.1,22.8)	
**Country income**	High			
High	13.2(10.2,16.3)	0.38	21.5(18.6,24.5)	0.001
Middle/low	17.2(8.2,26.1)		8.4(3.7,13.1)	

### Pooled estimates for biological and psychosocial risk factors

As shown in Table 4, child sociodemographic variables predictive of parental concerns on the *PEDS* indicating high developmental risk included male gender [14, 16, 17, 27, 28, 30, 37, 40, 47–50, 52, 54], age 3 years and above [14, 27, 28, 47–50], low birth weight [17, 37], poor/fair child health [40, 47–50] or special health care needs [16, 30]. Family sociodemographic variables predictive of parental concerns on the *PEDS* indicating high developmental risk included poor maternal mental health [7, 37, 40], low family SES [7, 16, 30, 40, 47–50], being of African American [7, 14, 17, 30, 47–50], Hispanic [7, 16, 17, 30, 47–50], First Nations and Australian Aboriginal ethnicity [14, 47–50, 54], being from a Non English speaking household [30, 47–50]. Service level variables predictive of parental concerns on the *PEDS* indicating high developmental risk included not having a usual source of health care/medical home [16, 30, 37, 40, 47, 49, 50]; or having public/no health insurance [7, 16, 30, 37, 47–50]. Parents not completing high school [16, 27, 28, 30, 40, 50] and single parenthood [16, 40, 47–50, 54] were significant using unadjusted OR, however not significant as adjusted OR [17, 37]. Children not being read to daily was significant in the unadjusted analysis [40, 47–49], however this did not appear to be significant in the one study that included it in a multivariate analysis (p = 0.93) [40]. Family size (more than 6 people in household) was not significant [47–50]. Parents of children who did not attend formal childcare were less likely to have concerns on the *PEDS* that indicated high developmental risk [40, 47–49], however findings from multivariate analysis of NSCH 2007 data aOR =1.05 (CI 0.84,1.33) found a non -significant effect of childcare and that receiving more than 10 hours a week of care at another family’s home was a risk factor (aOR = 1.71, p < 0.05) [17].

**Table 4:** **Risk factors associated with parental concerns on** ***PEDS*** **indicating high developmental risk**

Risk factor	N studies	Summary effect OR (95% CI)	P value	Heterogeneity (I ^2^)
**Child level**				
Male gender	11	1.68 (1.48,1.87)	<0.001	88.1%
Male gender (aOR)	3	2.01 (1.38, 2.94)	<0.001	91.6%
>3 years of age	7	1.83 (1.39,2.41)	<0.001	92.7%
Low birth weight	2	1.95 (1.53,2.49)	<0.001	0.0%
Poor/fair child health status	5	3.68 (2.66,5.08)	<0.001	68%
Special health care needs	2	4.86 (2.81,8.38)	<0.001	98%
**Family level**				
Poor maternal mental health (aOR)	3	1.61(1.31,1.99)	<0.001	0.0%
Low socioeconomic status	8	2.12(1.65,2.72)	<0.001	93.9%
Low socioeconomic status (aOR)	2	1.66 (1.08,2.53)	0.019	0.0%
Less than high school education	6	1.79 (1.14,2.79)	0.011	95.5%
Less than high school education (aOR)	3	1.94 (0.60,6.23)	0.26	98.8%
Being read to less than daily	4	1.47(1.28,1.67)	<0.001	30.6%
Family size (6 or more people in household)	4	1.18(0.83,1.68)	0.35	91.9%
Single parent	7	1.46 (1.16,1.84)	0.001	96.8%
Single parent (aOR)	2	0.94(0.75, 1.17)	0.57	0.0%
Ethnicity (vs White)				
African American	7	1.95 (1.43,2.66)	<0.001	90.5%
African American (aOR)	2	1.40 (1.10,1.78)	0.006	0.0%
Indigenous	6	1.98 (1.37,2.86)	<0.001	63.6%
Hispanic	4	2.24(1.83,2.72)	<0.001	88.9%
Hispanic (aOR)	2	1.54(1.16,2.04)	0.03	0.0%
Language spoken				
English as a second language (all)	5	1.61(1.12,2.33)	0.01	94.2%
English as a second language (Spanish)	5	1.48(0.87,2.53)	0.15	96.5%
**Service Level**				
No Usual Health Care/Medical Home(USA)	5	2.27(1.35,3.81)	0.002	84.3%
No or Public Health Insurance	4	1.85(1.45,2.37)	<0.001	95.2%
No Health Insurance (aOR)	2	2.01(1.01,4.02)	0.048	57.2%
Does not attend formal childcare	4	0.88(0.77,0.99)	0.040	0%

aOR = adjusted OR.

### Narrative summary of single studies, cumulative risk and life course analysis

A wide range of additional child, family, and service level factors were noted in single studies [36, 37, 39, 40, 56]. Child level factors were ear infections prior to age 2 (p < 0.001) [40], history of hospital admissions aOR 1.80 (95% CI 1.35–2.40) [37] and being underweight aOR 2.66 (95% CI 1.68–4.24) [37]. Family level factors were low scores on contentment/relaxation during pregnancy aOR 2.5 (95% CI 1.4 -4.2) [39], poor parenting morale when the child was 3 years old aOR 3.9 (95% CI 2.1- 7.3) [39], maternal history of domestic violence at pregnancy aOR 2.2 (95% CI 1.3- 3.7) [39, 40], household food-insecurity (aOR 1.76 (95% CI 1.26 - 2.46) [37], severe energy insecurity aOR 1.82 (95% CI 1.38 -2.39) [36], geographic site differences in the USA (p = 0.003) [37] and poor overall social support (p = 0.003) [39]. Service level factors were referral to early intervention (p < 0.001), speech pathology (p < 0.001) or audiology (p < 0.001) [40], lack of care coordination aOR 0.33 (95% CI 0.24–0.46), referrals aOR 0.40 (95% CI 0.25–0.65), family-centred care aOR 0.47 (95% CI 0.36–0.62) [30] and parental difficulty understanding the doctor uOR 3.35 (95% CI 2.1-5.4) [48].

Two studies reported a dose–response relationship between the number of risk factors and the increased likelihood of parental concerns on the *PEDS* indicating high developmental risk [7, 39, 40]. In one study having one risk factor was associated with an aOR 1.7 (95% CI: 1.20–2.38); two risk factors aOR 3.28, (95% CI: 2.27–4.73), three risk factors aOR 4.69 (CI: 2.84–7.73), and four risk factors aOR 14.58 (95% CI: 4.98–42.64) compared to a child with zero risk factors [7]. In addition, the greater the number of risk factors experienced by the child the more likely the child was to not receive comprehensive well child care [7].

The only longitudinal cohort in the review reported that at the second follow up when a child was 5 years of age male gender aOR 2.3 (1.3, 4.1), maternal history of abuse at pregnancy aOR 2.4 (1.3, 4.4) and poor parenting morale when the child was 3 years old aOR3.9 (2.1, 7.3) were predictors of parental concerns on the *PEDS* indicating high developmental risk [39].

## Discussion

### Prevalence and associated risk factors for parental concerns on the *PEDS*indicating developmental risk

This systematic review provides synthesised critically appraised evidence of the substantial global prevalence of parental concerns on the *PEDS* that indicate high and moderate developmental risk, which increases with biological and psychosocial adversity. This information is useful for researchers, service providers and clinicians to quantify the level of parental concern and to estimate the risk of children having developmental problems in the general population and to identify vulnerable subpopulations. Gender, low birth weight, poor maternal mental health, low family SES, minority ethnicity, speaking a language other than English and a lack of stimulation, such as a child not being read to, are all associated with adverse impacts on development in the literature and this was supported by the synthesised evidence [1, 60–63]. The increasing parental concerns with age of a child regardless of SES demonstrated in this review reflect the increasing developmental demands with age. The impact of child’s poor general health on developmental risk may reflect a true increase as some chronic illnesses and syndromes are associated with adverse developmental outcomes. However concerns about their child’s health may increase parental concerns generally [64, 65].

This review demonstrated that lack of access to usual and comprehensive health care in the USA and Canada was associated with an increased prevalence of parental concerns on the *PEDS* indicating high developmental risk. Interestingly the evidence for access to services such as early childhood education which has been found to particularly benefit children from disadvantaged backgrounds was not demonstrated [66–68].

Two studies demonstrated that parental concerns on the *PEDS* indicating high developmental risk increased with the number of risk factors a child was exposed to, consistent with our understanding of the burden of multiple risk factors on early childhood development [7, 39, 40, 62]. In addition, the “inverse care law” applied in one USA study, with the greater the number of risk factors, the less access to comprehensive health care [7, 69].

### Comparison with other measures of developmental risk

The confidence intervals around the pooled prevalence estimates of high and moderate developmental risk using the *PEDS* (27.0-36.0%) is similar to rates using the *Denver Developmental Screening Test (DDST)* [70–72] but higher than those using the *Australian Early Development Index (AEDI)* [1], and *Ages and Stages Questionnaire (ASQ)* [38, 43, 52]. While the *PEDS* gives an estimate of high and moderate developmental risk based on parental concerns this is not synonymous with a comprehensive developmental assessment. The *PEDS* specificities of 73-86% for parental concerns indicating high and/or moderate developmental risk means that some children identified by parental concern will be false positives [14, 17]. If parental concerns indicating only high developmental risk are examined the specificity of the *PEDS* improves to 89%, reducing the number of false positives but the sensitivity drops substantially to less than 50% giving an unacceptable level of false negatives [38, 43]. Thus, the true prevalence of actual developmental problems indicated by parental concerns is likely to lie somewhere between the values indicating high and moderate developmental risk [38, 73]. This is reflected in how the *PEDS* is used in clinical practice with those children identified as at high developmental risk on parental concerns referred on for a comprehensive developmental assessments and those at moderate risk undergoing a secondary developmental screen with a tool such as the *ASQ* and if they fail that then being referred on for a comprehensive developmental assessment [38, 43, 52]. Systematic reviews of the diagnostic test accuracy (DTA) of the tools that measure developmental risk such as the *PEDS* in relation to the reference-standard diagnostic batteries in nationally representative samples with an inclusive analysis of vulnerable subpopulations would be useful in understanding how useful developmental risk is as a way to estimate the burden of developmental problems in a population. This systematic review only included studies which had used the *PEDS*. Prevalence and DTA systematic reviews of other tools such as the *ASQ* and *AEDI* would also be useful for further comparison.

### Limitations

There was considerable variation in quality of the individual studies included in this systematic review. The major sources of bias were an inadequate sampling method, sampling frame, sample size and response rate and a lack of information to aid interpretation and applicability of the results including reporting of confidence intervals and subgroups. It is suggested that future research focus on designing studies that address these issues. Where community samples were used, parents most concerned about their children may be over-represented and this could lead to an overestimation of prevalence. However meta-regression using quality of the study as a variable did not find significant differences in pooled prevalence estimates. There were significant differences in pooled prevalence estimates of developmental risk between studies when the subgroups of publication type and country income were examined. We did not have individual patient data to undertake our own multivariate analysis. Although covariates were similar between studies, how these were measured and the breadth of variables measured varied. This highlights the need for agreed tools on measurement of psychosocial risk in research.

The cross sectional nature of the majority of papers in the review means that only associations of prevalence with risk factors can be examined not causality. In an attempt to address the issue of misclassification bias due to false positives we only examined the relationship between risk factors and high versus low/no developmental risk. Thus any significant relationship is likely to be an underestimate of the true strength of association [17].

## Conclusions

This systematic review found that the prevalence of parental concerns indicating developmental risk on the *PEDS* is substantial. As with most systematic reviews there were methodological issues with many of the primary studies with variable quality in sampling, representativeness and reporting. Nevertheless, the level of parental concerns that indicate developmental risk highlights the need to support families and promote early childhood development. At the individual level, parents, especially those in disadvantaged communities, should be asked systematically about their concerns and service providers should respond to these concerns through advice, support and facilitating further assessment and early intervention as required [11, 74, 75]. At the population level families should have access to universal high quality early childhood services that optimise child development. Given the prevalence of parental concerns increased with biological and psychosocial adversity, the service response needs to be one of proportionate universalism where the greater the disadvantage, the more services available [68].

## Electronic supplementary material

Additional file 1: **Search strategy.** (DOC 34 KB)

## Abbreviations

PEDS: Parents Evaluation of Developmental Status.

## References

[1] Goldfeld S, O’Connor M, Sayers M, Moore T, Oberklaid F (2012). Prevalence and correlates of special health care needs in a population cohort of australian children at school entry. J Dev Behav Pediatr.

[2] Guralnick MJ (1997). Effectiveness of early intervention for vulnerable children: a developmental perspective. Am J Ment Retard.

[3] King EH, Logsdon DA, Schroeder SR (1992). Risk factors for developmental delay among infants and toddlers. Child Health Care.

[4] Zeanah CH, Boris NW, Larrieu JA (1997). Infant development and developmental risk: a review of the past 10 years. J Am Acad Child Adolesc Psychiatry.

[5] Patrianakos-Hoobler AI, Msall ME, Marks JD, Huo D, Schreiber MD (2009). Risk factors affecting school readiness in premature infants with respiratory distress syndrome. Pediatrics.

[6] Sameroff A, Sameroff AE (2009). The Transactional Model Of Development: How Children And Contexts Shape Each Other. (pp. 3–21). xiv, 290 pp. The Transactional Model.

[7] Stevens GD (2006). Gradients in the health status and developmental risks of young children: the combined influences of multiple social risk factors. Matern Child Health J.

[8] Zuckerman B, Stevens GD, Inkelas M, Halfon N (2004). Prevalence and correlates of high-quality basic pediatric preventive care. Pediatrics.

[9] NH&MRC: Centre for Community Child Health (RCH) (2002). Child Health Screening and Surveillance: A Critical Review of the Literature.

[10] AAP (2006). Identifying infants and young children with developmental disorders in the medical home: an algorithm for developmental surveillance and screening. Pediatrics.

[11] Oberklaid F, Baird G, Blair M, Melhuish E, Hall D (2013). Children’s health and development: approaches to early identification and intervention. Arch Dis Child.

[12] National Screening Committee: **Child health sub-group report on developmental and behavioural problems.** http://www.screening.nhs.uk/policydb_download.php?doc=144

[13] Glascoe FP (1999). Using parents’ concerns to detect and address developmental and behavioral problems. J Soc Pediatr Nurs.

[14] Glascoe F (2013). Collaborating with Parents: Using Parents’ Evaluation of Developmental Status (PEDS) to Detect And Address Developmental And Behavioral Problems.

[15] Armstrong M, Goldfeld S (2004). Good Beginnings For Young Children And Families: A Feasibility Study.

[16] National Survey of Children’s Health, Data Resource Center for Child and Adolescent Health, Centre for Disease Controlhttp://childhealthdata.org/learn/NSCH accessed Sept2013

[17] Simon AE, Pastor PN, Avila RM, Blumberg SJ (2013). Socioeconomic disadvantage and developmental delay among US children aged 18 months to 5 years. J Epidemiol Community Health.

[18] Ng W, Reynolds D, Kennedy E, Feightner K, Holowaty P, Wade K, Fleiszer P, Northrup D, Glascoe F (2010). Measuring the prevalence of children at risk using; parents’ evaluation of developmental status in a telephone survey. Child Indicators Res.

[19] Loney PL, Chambers LW, Bennett KJ, Roberts JG, Stratford PW (1998). Critical appraisal of the health research literature: prevalence or incidence of a health problem. Chronic Dis Can.

[20] Agresti A, Coull B (1998). Approximate is better than ‘exact’ for interval estimation of binomial proportions. Amer Statist.

[21] Hamza T, van Houwelingen H, Stijnen T (2008). The binomial distribution of meta-analysis was preferred to model within-study variability. J Clin Epidemiol.

[22] Williams JG, Higgins JP, Brayne CE (2006). Systematic review of prevalence studies of autism spectrum disorders. Arch Dis Child.

[23] Moher D, Liberati A, Tetzlaff J, Altman D, The PRISMA Group (2009). Preferred reporting items for systematic reviews and meta-analyses: the PRISMA statement. PLoS Med.

[24] Chuan LB, Hock TT, Bin Bujang MA, Haniff J, Chang WS, Abdullah MR, F.P G (2012). Parents’ Evaluation Of Developmental Status – Validation And Feasbility Of Use Of Its Tranlsated Malay And Mandarin Version.

[25] Davies S, Feeney H (2009). A pilot of the Parents’ Evaluation of Developmental Status tool. Community Pract.

[26] Roux A, Herrera P, Wold C, Dunkle M, Glascoe F, Shattuck P (2011). Using 211 to Reach an Underserved Population with Developmental and Autism Screening. Washington University in St. Louis.http://www.pedstest.com/Research/tabid/91/articleType/CategoryView/categoryId/1/Articles-and-Abstracts.aspx accessed Jan 2013.

[27] Armstrong MF, Goldfeld S (2008). Systems of early detection in Australian communities: the use of a developmental concern questionnaire to link services. Aust J Adv Nurs.

[28] Coghlan D, King J, Wake M (2003). Parents’ Evaluation of Developmental Status in the Australian day-care setting:Developmental concerns of parents and carers. J Paediatr Child Health.

[29] Bethell C, Reuland C, Schor E, Abrahms M, Halfon N (2011). Rates of parent-centered developmental screening: disparities and links to services access. Pediatrics.

[30] Coker TR, Shaikh Y, Chung PJ (2012). Parent-reported quality of preventive care for children at-risk for developmental delay. Acad Pediatr.

[31] Sarmiento Campos JA, Squires J, Ponte J (2011). Universal developmental screening: preliminary studies in Galicia, Spain. Early Child Dev Care.

[32] Glascoe FP (1997). The importance of discussing parents’ concerns about development: do Parents Express Concerns Spontaneously?. Ambul Child Health.

[33] Glascoe FP, Macias M, Herrera P, Brixey S, Simpson P, Li S: **How do screening tests perform in settings serving at-risk populations?** *J Dev Behav Pediatr* in press,2010 PEDStest website, http://www.pedstest.com/Research/tabid/91/articleType/CategoryView/categoryId/1/Articles-and-Abstracts.aspx accessed Jan 2013

[34] Zuckerman KE, Boudreau AA, Lipstein EA, Kuhlthau KA, Perrin JM (2009). Household language, parent developmental concerns, and child risk for developmental disorder. Acad Pediatr.

[35] McGookin E, D’Sa V (2011). Developmental screening in a pediatric care practice. Med Health R I.

[36] Cook JT, Frank DA, Casey PH, Rose-Jacobs R, Black MM, Chilton M, DeCuba SE, Appugliese D, Coleman S, Heeren T, Berkowitz C, Cutts DB (2008). A brief indicator of household energy security: associations with food security, child health, and child development in us infants and toddlers. Pediatrics.

[37] Rose-Jacobs R, Black MM, Casey PH, Cook JT, Cutts DB, Chilton M, Heeren T, Levenson SM, Meyers AF, Frank DA (2008). Household food insecurity: associations with at-risk infant and toddler development. Pediatrics.

[38] Sices L, Stancin T, Kirchner L, Bauchner H (2009). PEDS and ASQ developmental screening tests may not identify the same children. Pediatrics.

[39] Tough SC, Siever JE, Benzies K, Leew S, Johnston DW (2010). Maternal well-being and its association to risk of developmental problems in children at school entry. BMC Pediatr.

[40] Tough SC, Siever JE, Leew S, Johnston DW, Benzies K, Clark D: **Maternal mental health predicts risk of developmental problems at 3 years of age: follow up of a community based trial.** *BMC Pregnancy Childbirth* 2008.,**8**(16)**:** 10.1186/1471-2393-8-16 PMC2396150 18460217

[41] Kiing JS, Low PS, Chan YH, Neihart M (2012). Interpreting parents’ concerns about their children’s development with the Parents Evaluation of Developmental Status: culture matters. J Dev Behav Pediatr.

[42] Kosht-Fedeyshin M (2006). Translation of the Parents’ Evaluation of Developmental Status (PEDS) Developmental Screening Tool for Identification of Developmental Delay in Children from Birth to Five Years of Age in the Karagwe District of Northwestern Tanzania, East Africa: a pilot study. Internet J Trop Med.

[43] Limbos MM, Joyce DP (2011). Comparison of the ASQ and PEDS in screening for developmental delay in children presenting for primary care. J Dev Behav Pediatr.

[44] Malhi P, Singh P (2002). Role of Parents’ Evaluation of Developmental Status in detecting developmental delay in young children. Indian Pediatr.

[45] Matibag R, Navarro J (2008). Use of Parents Evaluation of Developmental Status (PEDS) Questionnaire Among Pre-School Children aged 2–5 years old: Developmental Concerns of Parents and Teachers.

[46] Oreto J, Navarro J, Tanchanco L (2010). The use of the Filipino Parents’ Evaluation of Developmental Status (PEDS) Among Children aged 0–8 years old.

[47] **California Health Interview Survey** 2009.http://healthpolicy.ucla.edu/chis/

[48] **California Health Interview Survey** 2007.http://healthpolicy.ucla.edu/chis/

[49] **California Health Interview Survey** 2005.http://healthpolicy.ucla.edu/chis/

[50] **California Health Interview Survey** 2003.http://healthpolicy.ucla.edu/chis/

[51] Palarca CT (2008). The Role of Parents’ Evaluation of Developmental Status (PEDS) As A Screening Tool In The Early Detection Of Developmental Disabilities And Behavioural Disorders In Filipino Children Ages 0–96 Months Old In Gingoong City.

[52] Restall G, Borton B (2010). Parents’ concerns about their children’s development at school entry. Child Care Health Dev.

[53] Theeranate K, Chuengchitraks S (2005). Parent’s Evaluation of Developmental Status (PEDS) detects developmental problems compared to Denver II. J Med Assoc Thai.

[54] VSEHQ (2011). Outcomes For Victorian Children At School Entry. State And Local Findings From The School Entrant Health Questionnaire 2008.

[55] Wake M, Gerner B, Gallagher S (2005). Does parents’ evaluation of developmental status at school entry predict language, achievement, and quality of life 2 years later?. Ambul Pediatr.

[56] Ibironke JO, Friedman DS, Repka MX, Katz J, Giordano L, Hawse P, Tielsch JM (2011). Child development and refractive errors in preschool children. Optom Vis Sci.

[57] Tough S, Siever J, Benzies K, Leew S, Johnston DW (2006). Study Report: Screening For Developmental Problems Among Preschool-Aged Children. Calgary Children’s Initiative.

[58] Glascoe FP (1999). A method for deciding how to respond to parents’ concerns about development and behavior… including commentary by Oberklaid F. Ambul Child Health.

[59] Gustawan WI, Machfudz S (2010). Validity of parents’ evaluation of developmental status (PEDS) in detecting developmental disorders in 3–12 month old infants. Paediatr Indones.

[60] Mistry RS, Benner AD, Biesanz JC, Clark SL, Howes C (2010). Family and social risk, and parental investments during the early childhood years as predictors of low-income children’s school readiness outcomes. Early Childhood Res Q.

[61] Linver MR, Brooks-Gunn J, Kohen D (1999). Parenting behavior and emotional health as mediators of family poverty effects upon young low-birthweight children’s cognitive ability. Ann N Y Acad Sci.

[62] Walker SP, Wachs TD, Grantham-McGregor S, Black MM, Nelson CA, Huffman SL, Baker-Henningham H, Chang SM, Hamadani JD, Lozoff B, Gardner JM, Powell CA, Rahman A, Richter L (2011). Inequality in early childhood: risk and protective factors for early child development. Lancet.

[63] Braveman PA, Egerter SA, Woolf SH, Marks JS (2011). When do we know enough to recommend action on the social determinants of health?. Am J Prev Med.

[64] Smart D, Sanson A, Baxter J, Edwards B, Hayes A (2008). Home-To-School Transitions For Financially Disadvantaged Children. Book Home-To-School Transitions For Financially Disadvantaged Children (Editor ed.^eds.).

[65] Glascoe FP (1998). The value of ‘parents’ evaluations of developmental status’ in detecting and addressing children’s developmental and behavioral problems. Assess Eff Interv.

[66] Anderson LM, Shinn C, Fullilove MT, Scrimshaw SC, Fielding JE, Normand J, Carande-Kulis VG (2003). The effectiveness of early childhood development programs: a systematic review. Am J Prev Med.

[67] Shonkoff JP (2003). From neurons to neighborhoods: old and new challenges for developmental and behavioral pediatrics. J Dev Behav Pediatr.

[68] Marmot M (2010). Fair Society, Healthy Lives. The Marmot Review.

[69] Hart JT (1971). The inverse care law. Lancet.

[70] Miller JE (1998). Developmental screening scores among preschool-aged children: the roles of poverty and child health. J Urban Health.

[71] Moraes MW, Weber APR, Santos MCO, Almeida FA (2010). Denver II: evaluation of the development of children treated in the outpatient clinic of Project Einstein in the Community of Paraisópolis. Einstein (Säo Paulo).

[72] Nair MK, George B, Padma K, Potti N, Elizabeth KE, Jeyaseelan L (2009). Developmental Evaluation Clinic–CDC experience. Indian Pediatr.

[73] Blanchard LT, Gurka MJ, Blackman JA (2006). Emotional, developmental, and behavioral health of American children and their families: a report from the 2003 National Survey of Children’s Health. Pediatrics.

[74] Kemp L, Harris E, McMahon C, Matthey S, Vimpani G, Anderson T, Schmied V, Aslam H, Zapart S (2011). Child and family outcomes of a long-term nurse home visitation programme: a randomised controlled trial. Arch Dis Child.

[75] Zoritch B, Roberts I, Oakley A (2000). Day care for pre-school children. Cochrane Database Syst Rev.

[CR76] The pre-publication history for this paper can be accessed here:http://www.biomedcentral.com/1471-2431/14/231/prepub

