# Supplementary material for: A systematic review of the prevalence of parental concerns measured by the Parents’ Evaluation of Developmental Status (PEDS) indicating developmental risk
Source: BMC Pediatr. 2014 Sep 13;14:231. doi: 10.1186/1471-2431-14-231 (PMC4175611; doi:10.1186/1471-2431-14-231)
Supplement: Supplementary file 1 — Additional file 1: Search strategy. (DOC 34 KB) [file 12887_2014_1155_MOESM1_ESM.doc]

Additional file 1-SEARCH STRATEGY

Medline(adapted Embase, Psychinfo, CINAHL)

Database: Ovid MEDLINE(R) <1946 to October Week 1 2012>

Search Strategy:

1     Developmental Disabilities/

2     Human Development/

3     (Development$ adj3 (vulnerab$ or status)).tw.

4     exp Child Development/

5     school readiness

6     developmental stage

7     developmental disorders/

8   (Development$ adj3 milestone$).tw.

9   child$ development$.tw.

10     (developmental$ adj3 (deficienc$ or disabilit$ or disable$ or disorder$ or dysfunction$ or impair$)).tw.

11     or/1-10

12    Mass Screening/

13 Risk factor/

14 Risk assessment/

15 Risk/

16    Population Surveillance/

17     exp questionnaires/

18    health status indicators/

19     Psychological Tests/

20 socioeconomic factors/

21    (development$ adj5 (index$ or indices or indicator$ or questionnaire$ or measure$ or surveillance or screen$ or test$)).tw.

22     (parent$ evaluation or ASQ or Denver or Brigance or Bayleys or Batelle or LSAC or AEDI).tw.

23     exp infant/

24    Child, Preschool/

25    (baby or babies or child$ or infant$ or preschool$ or pre-school$ or kindergarten$ or kinder-garten$ or nursery).tw.

26   23 or 24 or 25

27     or/12-22

28     epidemiologic studies/ or cross-sectional studies/ or prospective studies/

29     (cross-section$ adj3 (study or studies)).tw.

30   prevalence.tw.

31    prevalence/

32 cohort analysis/or controlled study/or logistic models/

33     or/28-32

35 11 and 27 and 33

**LILACS**

child and development and screening

**PUBMED**

Child development AND prevalence AND screening

**Cochrane Library**

“child development”

**Proquest**

child development and developmental screening/Child development and (developmental surveillance)/PEDS/Brigance/ASQ/Bayleys/Battelle/Denver/LSAC /AEDI

**ERIC**

(child development) AND all((developmental screening OR developmental surveillance))
